# Cellulose crystallinity index: measurement techniques and their impact on interpreting cellulase performance

**DOI:** 10.1186/1754-6834-3-10

**Published:** 2010-05-24

**Authors:** Sunkyu Park, John O Baker, Michael E Himmel, Philip A Parilla, David K Johnson

**Affiliations:** 1 Biosciences Center, National Renewable Energy Laboratory, 1617 Cole Blvd, Golden, CO 80401, USA; 2 National Center for Photovoltaics, National Renewable Energy Laboratory, 1617 Cole Blvd, Golden, CO 80401, USA; 3 Department of Forest Biomaterials, North Carolina State University, Raleigh, NC 27695, USA

## Abstract

Although measurements of crystallinity index (CI) have a long history, it has been found that CI varies significantly depending on the choice of measurement method. In this study, four different techniques incorporating X-ray diffraction and solid-state ^13^C nuclear magnetic resonance (NMR) were compared using eight different cellulose preparations. We found that the simplest method, which is also the most widely used, and which involves measurement of just two heights in the X-ray diffractogram, produced significantly higher crystallinity values than did the other methods. Data in the literature for the cellulose preparation used (Avicel PH-101) support this observation. We believe that the alternative X-ray diffraction (XRD) and NMR methods presented here, which consider the contributions from amorphous and crystalline cellulose to the entire XRD and NMR spectra, provide a more accurate measure of the crystallinity of cellulose. Although celluloses having a high amorphous content are usually more easily digested by enzymes, it is unclear, based on studies published in the literature, whether CI actually provides a clear indication of the digestibility of a cellulose sample. Cellulose accessibility should be affected by crystallinity, but is also likely to be affected by several other parameters, such as lignin/hemicellulose contents and distribution, porosity, and particle size. Given the methodological dependency of cellulose CI values and the complex nature of cellulase interactions with amorphous and crystalline celluloses, we caution against trying to correlate relatively small changes in CI with changes in cellulose digestibility. In addition, the prediction of cellulase performance based on low levels of cellulose conversion may not include sufficient digestion of the crystalline component to be meaningful.

## Background

Cellulose is a high molecular weight linear polymer composed of D-glucopyranose units linked by β-1,4-glycosidic bonds. The repeating unit of cellulose is cellobiose. Hydroxyl groups present in cellulose macromolecules are involved in a number of intra- and intermolecular hydrogen bonds, which result in various ordered crystalline arrangements. Four different crystalline allomorphs have been identified by their characteristic X-ray diffraction (XRD) patterns and solid-state ^13^C nuclear magnetic resonance (NMR) spectra: celluloses I, II, III and IV. Cellulose I is the most abundant form found in nature. Cellulose II can be prepared by two distinct routes: mercerization (alkali treatment) and regeneration (solubilization and subsequent recrystallization). Celluloses III_I _and III_II _can be formed from celluloses I and II, respectively, by treatment with liquid ammonia, and the reaction is reversible [1]. Celluloses IV_I _and IV_II _can be obtained by heating celluloses III_I _and III_II_, respectively [2]. Thorough reviews of cellulose crystalline allomorphs can be found elsewhere [3-5].

The crystalline structure of cellulose has been studied since its discovery in the 19th century. Currently, cellulose I is receiving increased attention due to its potential use in bioenergy production. The crystalline structure of cellulose was first established by Carl von Nägeli in 1858 [6], and the result was later verified by X-ray crystallography [7]. Several different models of cellulose I have been proposed since then; however, its structure is still not fully understood because of its complexity. It is known that the crystalline structure of cellulose I is a mixture of two distinct crystalline forms: celluloses I_α _(triclinic) and I_β _(monoclinic), which were verified using solid-state ^13^C NMR [8]. The relative amounts of celluloses I_α _and I_β _vary with the source of the cellulose, with the I_β _form being dominant in higher plants. The size of cellulose crystallites is small, generally about 5 nm in width, thus the resolution of the XRD pattern is not sufficient to extract exact information about crystal lattices within the structure. Cellulose crystallites are thought to be imperfect, and thus a significant portion of the cellulose structure is less ordered; this portion is often referred to as amorphous. A parameter termed the crystallinity index (CI) has been used to describe the relative amount of crystalline material in cellulose. The traditional two-phase cellulose model describes cellulose chains as containing both crystalline (ordered) and amorphous (less ordered) regions [9].

The CI of celluloses have been measured using several different techniques including XRD, solid-state ^13^C NMR, infrared (IR) spectroscopy and Raman spectroscopy. There have also been several methods used for calculating CI from the raw spectrographic data, particularly for XRD. Methods using Fourier transform (FT)-IR spectroscopy determine CI by measuring relative peak heights or areas [10-12]. The determination of CI using FT-IR spectroscopy is the simplest method, but gives only relative values, because the spectrum always contains contributions from both crystalline and amorphous regions. In many studies, the CI calculated from an FT-IR spectrum is compared with those from XRD and/or NMR measurements. Because the FT-IR method is not an absolute measurement technique, we chose not to use it in this study. Raman spectroscopy has also been employed to determine CI [13].

The CI of cellulose has been used for more than five decades to interpret changes in cellulose structure after physicochemical and biological treatments. However, it has been found that the CI varies significantly, depending on the choice of measurement method [11,14,15]. Thygesen and co-workers compared four different analysis techniques involving XRD, and reported that the CI of Avicel cellulose varied significantly from 39% to 67%, depending on the technique used [15].

In this study, we made critical comparisons between the different techniques using XRD and solid-state ^13^C NMR. Comparisons were made with literature data for the CI of one type of cellulose (Avicel PH-101) using these methods. In addition, we measured the CI of eight celluloses from different sources to demonstrate the dissimilarity in results that can be obtained using different methods. The effect of interpreting cellulose enzymatic digestibility in terms of the crystallinities determined by the different techniques is also discussed.

## Materials and methods

### Cellulose samples

Eight high purity (>95% cellulose in all cases except for Solka-Floc, which was >93%) celluloses were used in this study. Bacterial microcrystalline cellulose (BMCC) was prepared from *Gluconacetobacter hansenii *(American Type Culture Collection (ATCC) 10821) in our laboratory [16]. The seven other celluloses were commercially available: Sigmacell 50 (S5504), Sigmacell 20 (S3504), Avicel PH-101 (11365), Fluka cellulose (22183), α-cellulose (C8002) (all purchased from Sigma-Aldrich, St. Louis, MO, USA), Solka-Floc (International Fiber Corporation (North Tonawanda, NY, USA) and JT Baker cellulose (1529) (Mallinckrodt Baker, Phillipsburg, NJ, USA). Ball milled cellulose was prepared by milling Avicel PH-101 (1.5 g) for 20 minutes in a cryogenic impact mill (6770 Freezer Mill; Spex, Metuchen, NJ, USA) cooled by liquid nitrogen.

### CI of celluloses

**Figure 1:**
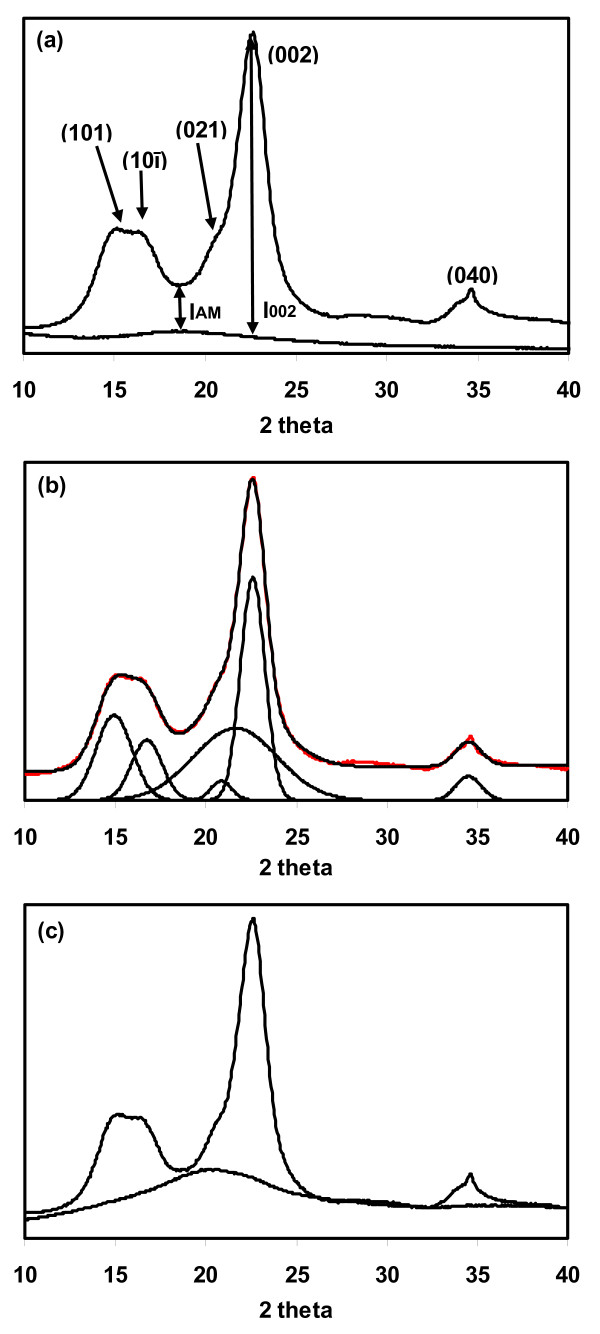
**X-ray diffraction spectra of Avicel PH-101 illustrating the three most common methods for calculating CI**. **(a) **Peak height method, **(b) **peak deconvolution method and **(c) **amorphous subtraction method. The XRD data were collected using CuKα radiation.

The CI of the eight celluloses was measured by two different techniques: XRD and solid-state ^13^C NMR. XRD was performed on a four-circle goniometer (XDS-2000 Polycrystalline Texture Stress (PTS) goniometer; Scintag, Scintag Inc., Cupertino, CA, USA) using CuKα radiation generated at 45 kV and 36 mA. The CuKα radiation consists of Kα1 (0.15406 nm) and Kα2 (0.15444 nm) components, and the resultant XRD data has both components present; the CuKα radiation is filtered out from the data using a single-channel analyzer on the output from the semiconductor detector, and does not contribute to the data. The source slits were 2.0 mm and 4.0 mm at a 290 mm goniometer radius, and the detector slits were 1.0 mm and 0.5 mm at the same radius. Dried cellulose samples (approximately 0.5 g) were mounted onto a quartz substrate using several drops of diluted glue. This diluted glue is amorphous when it is dry, and adds almost no background signal (lower line in Figure 1a). Scans were obtained from 5 to 50 degrees 2θ in 0.05 degree steps for 15 seconds per step.

**Figure 2:**
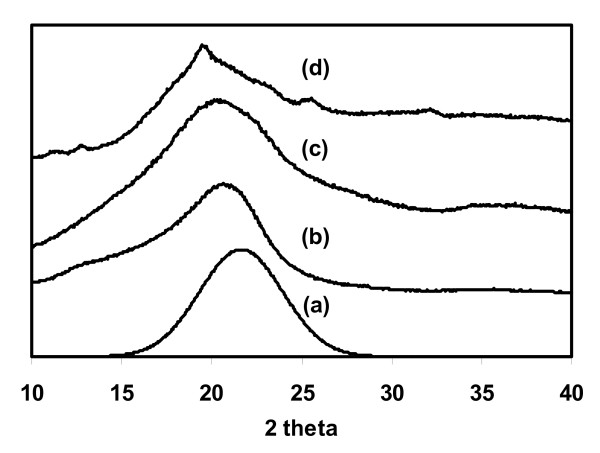
**X-ray diffraction spectra of amorphous cellulose examples**. **(a) **Amorphous portion extracted by the peak deconvolution method (Figure 1b), **(b) **amorphous cellulose produced by the DMSO/PF method [70], **(c) **ball-milled cellulose and **(d) **commercial xylan (oak spelt xylan, Aldrich 36355-3).

To calculate the CI of cellulose from the XRD spectra, three different methods were used. First, CI was calculated from the height ratio between the intensity of the crystalline peak (I_002 _- I_AM_) and total intensity (I_002_) after subtraction of the background signal measured without cellulose [17-19] (Figure 1a). Second, individual crystalline peaks were extracted by a curve-fitting process from the diffraction intensity profiles [20,21]. A peak fitting program (PeakFit; www.systat.com) was used, assuming Gaussian functions for each peak and a broad peak at around 21.5° assigned to the amorphous contribution (Figure 1b). Iterations were repeated until the maximum F number was obtained. In all cases, the F number was >10,000, which corresponds to a R^2 ^value of 0.997. Third, ball-milled cellulose (Figure 2c) was used as amorphous cellulose to subtract the amorphous portion from the diffraction profiles [15] (Figure 1c). After subtracting the diffractogram of the amorphous cellulose from the diffractogram of the whole sample, the CI was calculated by dividing the remaining diffractogram area due to crystalline cellulose by the total area of the original diffractogram.

**Figure 3:**
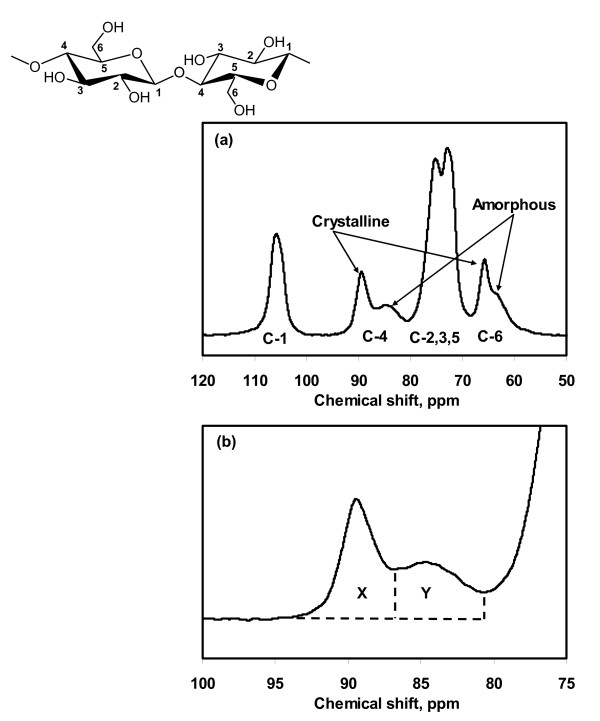
**Solid state ^13^C NMR spectrum of Avicel PH-101**. **(a) **Whole spectrum showing the assignment of peaks to the carbons in a glucopyranose repeat unit and **(b) **sub-spectrum showing peaks assigned to the C4 in cellulose. The CI is calculated by *x*/(*x*+*y*).

Solid-state ^13^C NMR spectra were collected at 4.7 T with cross-polarization and magic angle spinning (MAS) in a 200 MHz spectrometer (Avance; Bruker, Madison, WI, USA). Variable amplitude cross-polarization was used to minimize intensity variations of the non-protonated aromatic carbons that are sensitive to Hartmann-Hahn mismatch at higher MAS rotation rates [22]. The ^1^H and ^13^C fields were matched at 53.6 kHz, and a 1 dB ramp was applied to the proton rotating-frame during the matching period. Acquisition time was 0.051 seconds, and sweep-width was 20 kHz. MAS was performed at 6500 Hz. The number of scans was 10,000 to 20,000 with a relaxation time of 1.0 seconds. The CI was determined by separating the C4 region of the spectrum into crystalline and amorphous peaks, and calculated by dividing the area of the crystalline peak (87 to 93 ppm) by the total area assigned to the C4 peak (80 to 93 ppm) [23] (Figure 3a, Figure 3b).

## Results and discussion

XRD and solid-state ^13^C NMR have most widely been used to evaluate the CI of cellulose and the spectral analysis techniques that have been used are summarized here. Figure 1a shows the XRD spectrum of Avicel PH-101, with the peaks labeled to indicate their crystal lattice assignments, assuming the I_β_ phase is aligned with the fiber axis along the *b *direction [24]. Figure 3a shows the solid-state ^13^C NMR spectrum of Avicel PH-101; the labels show which peaks have been assigned to the different carbon atoms of the glucopyranose repeating units in cellulose, and which peaks have been attributed to the carbon atoms in crystalline and amorphous cellulose.

For the XRD methods, one important factor to consider is the preferred orientation of the crystallites (also known as texture). Often the manner in which samples are synthesized, the nature of the crystallites and the method of sample preparation for XRD causes the development of texture in the sample. It is well known that this will drastically influence the relative intensities of the diffraction peaks and will correspondingly influence the CI. How much this influence extends depends on the exact definition of the CI. The best suggestion to avoid a texture-biased CI is to carefully prepare samples to eliminate or minimize texture [15].

In its present state, measurement of cellulose CI by XRD provides a qualitative or semi-quantitative evaluation of the amounts of amorphous and crystalline cellulosic components in a sample. Development of a truly quantitative cellulose CI is laudable, but would need to proceed along the principles established for quantitative XRD phase analysis. [25,26]. The greatest barrier to this goal is the lack of appropriate cellulose standards needed to calibrate the measurement. Most current cellulose CI definitions do not follow such principles.

### Method 1: the XRD peak height method

This method, developed by Segal and coworkers [19], examined the changes in XRD spectra during decrystallization of cotton cellulose by chemical and mechanical methods. The proposed method was for empirical measurements to allow rapid comparison of cellulose samples. CI was calculated from the ratio of the height of the 002 peak (I_002_) and the height of the minimum (I_AM_) between the 002 and the 101 peaks, as shown in Figure 1a. This method is useful for comparing the relative differences between samples; however, we suggest that it should not be used as a method for estimating the amount of crystalline and amorphous material in a cellulose sample for the following reasons.

1. The minimum position between the 002 and the 101 peaks (I_AM _which is at about 18.3° in Figure 1a) is not aligned with the maximum height of the amorphous peak. The apex of the peak that is due to amorphous cellulose is likely to be higher than 18.3°. As shown in Figure 2, the apex of the peak of regenerated amorphous cellulose (2b) was found to be at 20.7°, ball milled cellulose (2c) was at 20.5° and commercial xylan (2d) was at 19.5°. From the peak deconvolution method, the amorphous peak (2a) was predicted to be at around 21.5°. Thus, the I_AM _value for the height method is significantly underestimated, resulting in an overestimation of the CI.

2. There are at least four crystalline peaks, but only the highest peak (002) is used in the calculation. This excludes contributions from the other crystalline peaks, putting too much emphasis on the contribution from one alignment of the cellulose crystal lattice.

3. Peaks in the cellulose diffraction spectrum are very broad and vary considerably in their width. A simple height comparison cannot be expected to provide a reasonable estimate of cellulose crystallinity, as it neglects variation in peak width, which can also be affected by crystallite size [21].

We believe that for these reasons the relative height to the minimum can only be taken as a rough approximation of the contribution of amorphous cellulose to the cellulose diffraction spectrum.

### Method 2: the XRD deconvolution method

This method requires software to separate amorphous and crystalline contributions to the diffraction spectrum using a curve-fitting process. For the curve fitting, a few assumptions have to be made, such as the shape and number of peaks. Gaussian [20,27], Lorentzian [14] and Voigt [21] functions are commonly used for deconvolution of XRD spectra. Five crystalline peaks (101, 10ī, 021, 002 and 040) have been separated in many cases [20,21], but four crystalline peaks (101, 10ī, 002 and 040) have been assumed in other studies [14]. Figure 1b shows the deconvolution of Avicel PH-101 using five Gaussian crystalline peaks. CI is calculated from the ratio of the area of all crystalline peaks to the total area.

An important assumption for this analysis is that increased amorphous contribution is the main contributor to peak broadening. However, in addition to crystalline disorder (amorphous content), there are other intrinsic factors that influence peak broadening, such as crystallite size and non-uniform strain within the crystal. It might be possible to deconvolute these contributions with well-behaved samples that can be resolved into many narrow diffraction peaks over a significant range of 2θ. Unfortunately, cellulose peaks are very broad and not well resolved, with overlapping peaks. It is generally accepted in the cellulose community that peak broadening is due to the amorphous cellulose. However, crystallite size is an equally important issue for peak broadening and some studies have assumed that the latter was the main contributor [21]. Information about average crystallite size has been calculated from this method using the Scherrer formula. The width of the crystalline peak (002) at half height has been directly related to crystallite size and calculated to be about 4 to 7 nm in most references [14,17,21,28].

### Method 3: the XRD amorphous subtraction method

The basis for this method was outlined by Ruland [29], who determined crystallinity by subtracting the amorphous contribution from diffraction spectra using an amorphous standard. The challenge is to select an amorphous standard that is similar to the amorphous component in the sample. Various materials have been used as an amorphous standard, such as ball-milled cellulose, regenerated cellulose, and xylan or lignin powder. A scale factor is applied to the spectrum of the amorphous material so that after subtraction of the amorphous spectrum from the original spectrum, no part of the residual spectrum contains a negative signal. Figure 1c shows how an amorphous spectrum has been scaled to just touch the diffraction spectrum to give the resulting subtracted spectrum that is due to the crystalline cellulose present in the sample. CI is calculated as the ratio between the area of the crystalline contribution and the total area.

### Method 4: the NMR C4 peak separation method

We have used solid-state ^13^C NMR to evaluate the CI of cellulose samples, employing the method of Newman [23]. In the NMR spectra in Figure 3, the peak at 89 ppm is assigned to the C4 carbon in ordered cellulose structures, and the peak at 84 ppm is assigned to the C4 carbon of disordered cellulose [30]. CI is calculated by dividing the area of the crystalline peak (integrating the peak from 87 to 93 ppm) by the total area assigned to the C4 peaks (integrating the region from 80 to 93 ppm). This approach has been used by others assessing the influence of cellulose crystallinity on cellulose digestibility [31].

This method was chosen over a more detailed analysis of the C4 peaks using peak deconvolution software because it was our goal is to determine the effect of CI on the digestibility of biomass derived celluloses, which have a relatively low order. As noted by Larsson [32], the lack of spectral detail in celluloses of low order make detailed analysis impossible. Peak deconvolution methods have been applied to more ordered celluloses [32]. The shape and number of peaks were selected so that they agreed with the mixed or composite crystal model of Atalla and VanderHart [8]. Lorentzian [33] and Gaussian [34-36] functions were used to perform the deconvolution of the C4 peaks. In some studies [37,38], a combination of Lorentzian and Gaussian functions was used to fit the C4 region (80 to 93 ppm) with seven peaks that range in full width at half height from 70 to more than 500 Hz. Compared with the detailed peak deconvolution methods, the Newman method incorporates the two peaks previously assigned to the fibril surface and the majority of the broad peak assigned to amorphous cellulose into the peak for disordered cellulose at 84 ppm. The peak assigned to more ordered cellulose structures (89 ppm) includes those peaks previously assigned to the I_α_, I_β _and paracrystalline cellulose components.

### Frequencies of methods and variations in the CI of Avicel PH-101

Based on a literature survey of about 80 journal articles that reported the CI for commercially available celluloses, the XRD peak height method is the most widely used to determine CI, being used in about 70 to 85% of the studies (Table 1). It seems likely that the popularity of this method results from its ease of use. The other methods were each used in 5 to 10% of the references found in this study. The XRD peak deconvolution method is widely used to analyze cellulose II structure, for example, in cellulose film and lyocell, because the XRD peak height method cannot be applied to the cellulose II allomorph. A typical X-ray diffraction profile of cellulose II can be found elsewhere [39].

**Table 1:** Frequencies of different methods reported in the literature for measuring the crystallinity index of commercial celluloses.

Instrument	Analysis technique	Frequency, %
XRD	Peak height	70 to 85

	Peak deconvolution	5 to 10

	Amorphous subtraction	5 to 10

NMR	C4 peak separation	5 to 10

NMR, nuclear magnetic resonance; XRD, X-ray diffraction.

About 80 articles (30 for Avicel PH-101 and 50 for other commercial celluloses) were analyzed in this study.

**Figure 4:**
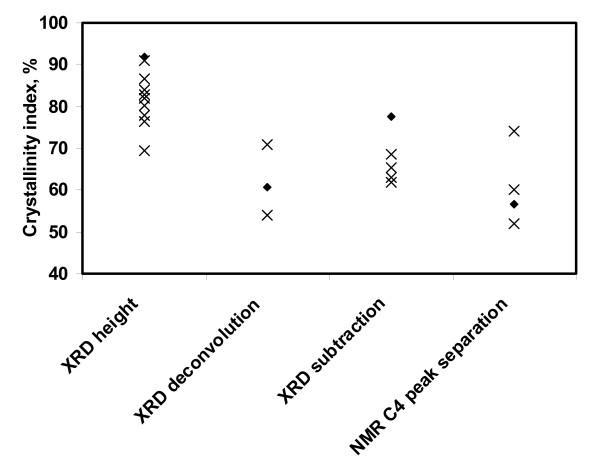
**CI of Avicel PH-101 from the literature in terms of measurement techniques**. Crosses indicate literature values and black diamonds indicate the values obtained by the authors.

Figure 4 shows the literature values for the CI of Avicel PH-101 categorized by the different measurement techniques, and it is obvious that the CI of cellulose measured by different methods produces different results. Avicel PH-101 was chosen because it was the most widely measured cellulose reported in the literature. We made the assumption that all Avicel PH-101 used in the literature was of the same quality, even though it has been reported that the quality of Avicel PH-101 can vary between batches and production locations [40,41].

The values plotted in Figure 4 were reported by several research groups (XRD peak height method [42-50], XRD deconvolution method [27,51], XRD amorphous subtraction method [40,52,53] and NMR C4 peak separation method [13,27,54]). The filled diamonds in Figure 4 are the values we obtained using the various techniques.

### CI of commercial celluloses

To demonstrate the differences in CI measured by different methods, eight cellulose samples were tested (Table 2). The BMCC sample gave the highest CI, and α-cellulose the lowest. Although the methods give different CI results for a given cellulose, the order of crystallinity for these celluloses is relatively consistent within each measurement technique. These results again show that the XRD peak height method produces a higher CI than the other methods. We found the value for Avicel PH-101 to be 91.7% using the XRD peak height method after baseline subtraction of the spectrum. Some of the reference values in Figure 4 were calculated without considering the baseline; our value would be 81.0% if calculated without baseline subtraction.

**Table 2:** CI of celluloses determined by four different methods by the authors.

Cellulose tested	XRD method			NMR method
	
	Peak height	Peak deconvolution	Amorphous subtraction	C4 peak separation
BMCC	95.2	73.1	82.4	73.8

Avicel PH-101*	91.7 ± 1.5	60.6 ± 1.0	77.7 ± 1.9	56.7 ± 2.2

SigmaCell 50	91.2	61.3	79.4	56.1

SigmaCell 20	84.8	64.2	67.0	52.6

JT Baker cellulose	85.5	61.5	69.1	49.1

Fluka cellulose	82.9	52.9	61.6	48.6

SolkaFloc cellulose	78.3	56.8	57.2	43.9

Sigma α-cellulose	78.0	55.9	54.4	41.5

BMCC, bacterial microcrystalline cellulose.

Values are means.

* For Avicel PH-101 the standard deviation from three measurements is given.

Generally, the different methods produce CI values in the following order: XRD height method > XRD amorphous subtraction > XRD peak deconvolution > NMR C4 peak separation. The important question is which method provides the most accurate evaluation of cellulose crystallinity. Because of the limitations and problems mentioned earlier, there is no simple answer. In addition, the structure of cellulose is still not fully understood and the assumption that cellulose has only two regions, crystalline and amorphous, might be not realistic. Some researchers have suggested that there is a paracrystalline region in cellulose, which is less ordered with a somewhat larger mobility than the crystalline cellulose structure [32].

### Interpretation of enzymatic hydrolysis of cellulose in terms of cellulose CI

Cellulose crystallinity has long been thought to play an important role in enzymatic hydrolysis [55]. The concept that cellulose structure is divided into two regions, an amorphous region that is easy for enzymes to digest and a crystalline region that is difficult to digest, is extremely appealing. This provides a ready explanation of observed cellulose digestion kinetics, where enzymes more rapidly digest the 'easy and presumed amorphous' material before more slowly digesting the more difficult crystalline cellulose. However, the interpretation of data on cellulose hydrolysis by enzymes in terms of the CI of the substrate is not straightforward, for several reasons.

First, the reported changes in CI after enzymatic hydrolysis do not show a clear trend. Even though many studies have produced evidence to support the idea that CI increases during enzymatic hydrolysis [18,56,57], the reported increase has often been small. Chen and co-workers [56] found only a 2.6% increase in CI after 18% conversion of bacterial cellulose. Wang and co-workers [57] found only a 2.0% increase in CI after 6 days of crude cellulase hydrolysis of cotton fibers. This suggests a slightly preferential hydrolysis of amorphous cellulose. In one case, it was reported that there was no discernible difference in the CI of hemp fibers [58] and unbleached kraft pulp [59], after partial enzymatic digestion. Thus, it is unclear from these data if there is a preferential digestion of the amorphous cellulose component. By contrast, celluloses that are made highly amorphous by dissolution in a cellulose solvent followed by regeneration have been shown to have extremely high hydrolysis rates, with initial rates approximately three times higher than those of untreated celluloses[60].

A second problem is the coupling of crystallinity with other cellulose properties. During any chemical/mechanical/biological treatment, the CI of cellulose can be changed and then correlated with the measured digestibility. However, differences in observed enzyme hydrolysis kinetics may be governed by other characteristics such as available surface area, degree of polymerization and particle size. For example, the increased digestibility for finely ground sawdust particles may be due to both decreased CI and increased surface area [61]. Decoupling CI from changes in other properties has proven extremely difficult [62].

A third problem is that the structure of cellulose is actually more complicated than the two-phase model (crystalline and amorphous) indicates. As mentioned, Larsson and co-workers [32] reported that the amount of paracrystalline cellulose (33.1%) is almost identical to the amount of crystalline structure (31.8%) in cotton cellulose. The existence of this transition region between crystalline and amorphous structures makes interpretation even more difficult. In addition, structural and enzymatic studies [63-66] on various celluloses have suggested that larger scale structures in celluloses may significantly affect the accessibility of cellulose to enzymes. For example, if an amorphous region is buried in the interior of a particle that is packed sufficiently tightly by neighboring crystallites to be essentially impenetrable to the enzymes, reaction with the amorphous component will probably be impeded.

A fourth problem is related to the measurement technique, especially for XRD measurements. From the literature survey, we found that a significant number of references for the XRD methods used spectra of very poor quality. To evaluate small changes in CI, it is crucial to have XRD with a high signal to noise level, as exemplified by the spectra shown here. None of the figures in this paper have been processed using a smoothing function; all show unprocessed raw data.

Finally, there are large discrepancies in the amorphous contents measured by different groups (between about 8 and 40% for Avicel PH-101, depending on method) and the thresholds at which cellulose digestion rates are reported to slow down. Andersen and co-workers [67] reported that when digested with a commercial enzyme mixture, Avicel PH-101 was hydrolyzed up to 7% in 24 hours. Most of this hydrolysis (5%) was accomplished in the first 5 hours of the digestion, with the hydrolysis rate decreasing sharply thereafter. Using single endoglucanases and cellobiohydrolases for the hydrolysis of Avicel PH-101, rather than complete systems, Szijártó and co-workers [68] found that each digestion curve showed a sharp decrease in rate at a point well below 2% conversion. Even earlier, Tomme and co-workers [69], studied the relationship between the hydrolytic capabilities of different cellulases with amorphous and crystalline Avicel. From their results on the crystalline substrate it can be estimated that cellulose conversion was <1.3% using an intact cellobiohydrolase (CBH) I from *Trichoderma reesei *and <0.2% for its proteolytically cleaved catalytic domain alone (both forms of the enzyme were used at a moderate but realistic loading ratio of 15 mg enzyme per gram of cellulose) during the three hour assay. In all cases, it appears that the amount of rapidly digested cellulose is substantially less than the amorphous cellulose content measured by any of the methods.

## Conclusions

It is clear that the most popular method for estimating cellulose CI, the XRD height method, produces values that are significantly higher than the other methods. Literature data for Avicel PH-101 and data from our measurement of eight other celluloses using four methods support this idea. The other methods studied in this work rank the celluloses in roughly the same order as the XRD height method; however, the CI values from the height method are significantly higher. It seems likely that the reason for the popularity of the XRD height method is that it is the easiest to use. It should be remembered that Segal and coworkers only intended this method to be used as a 'time-saving empirical measure of relative crystallinity' [19]. We suggest that the other XRD and NMR methods presented here, which consider the contributions from both amorphous and crystalline cellulose to the whole of the XRD or NMR spectrum, provide a more accurate measure of the crystallinity of cellulose samples.

Although celluloses having a high amorphous content are usually more easily digested by enzymes, it is unclear based on the studies published in the literature that CI provides a clear indication of the digestibility of a cellulose sample. Accessibility of plant cell wall cellulose microfibrils to the various exo- and endocellulases necessary for cellulose hydrolysis appears to be the most important factor in determining hydrolysis rate. Enzyme accessibility should be affected by crystallinity, but it is also known to be affected by the lignin and hemicellulose contents/distribution, the particle size, and the porosity of the native cell wall sample. Consequently, CI is just one of several parameters that should be considered in assessing the likely enzymatic hydrolysis rate of cellulose in a biomass sample. In addition, if the enzymes work ablatively on cellulose microfibril surfaces, consuming the less ordered surface layers of cellulose, then internal ordered cellulose chains will become surface chains with decreased order, so that conversion of 'amorphous cellulose' results in production of more 'amorphous cellulose' and a further decrease in cellulose CI.

Enzymatic cellulose hydrolysis is a complex process, and CI alone may not adequately explain differences in observed hydrolysis rates. Given the method dependency for determining the CI values of cellulose preparations likely to be used in assessing the performance of cellulases, and the complex nature of the interaction of cellulases with amorphous and crystalline celluloses, we caution against trying to correlate relatively small changes in CI with changes in cellulose digestibility. Similarly, it is difficult to interpret enzymatic cellulose digestion rate studies unless the digestion is taken near to completion, as it is unclear whether or not the enzyme has been acting on the more easily converted amorphous component. If the digestion is taken to completion, or at least to a level well beyond the amorphous content, uncertainty about the performance of the enzyme is reduced.

## Competing interests

The authors declare that they have no competing interests.

## Authors' contributions

JP obtained X-ray powder diffraction spectra on the various cellulose samples, calculated CI values from all NMR and XRD spectra by the various techniques, studied and analyzed the literature measurements of CI and drafted the manuscript. JOB contributed literature information on the relationship between enzymatic digestibility and CI and helped draft the manuscript. MEH helped draft the manuscript. PAP helped draft the manuscript and provided input on the measurement of CI by XRD. DKJ contributed to the original conception of the study, advised on the design and progress of the experimentation and helped draft the manuscript. All authors critically revised the draft and approved the final manuscript.
